# Supplementary material for: Splice-Junction-Based Mapping of Alternative Isoforms in the Human Proteome
Source: Cell Rep. Author manuscript; Available in PMC 2020 Jan 15. (PMC6961840; doi:10.1016/j.celrep.2019.11.026)
Supplement: 2 [file NIHMS1546469-supplement-2.zip › DF1/PXD010154/Heart/Heart_40_MYBPC3_RTDSHEDTGILDF.pdf]

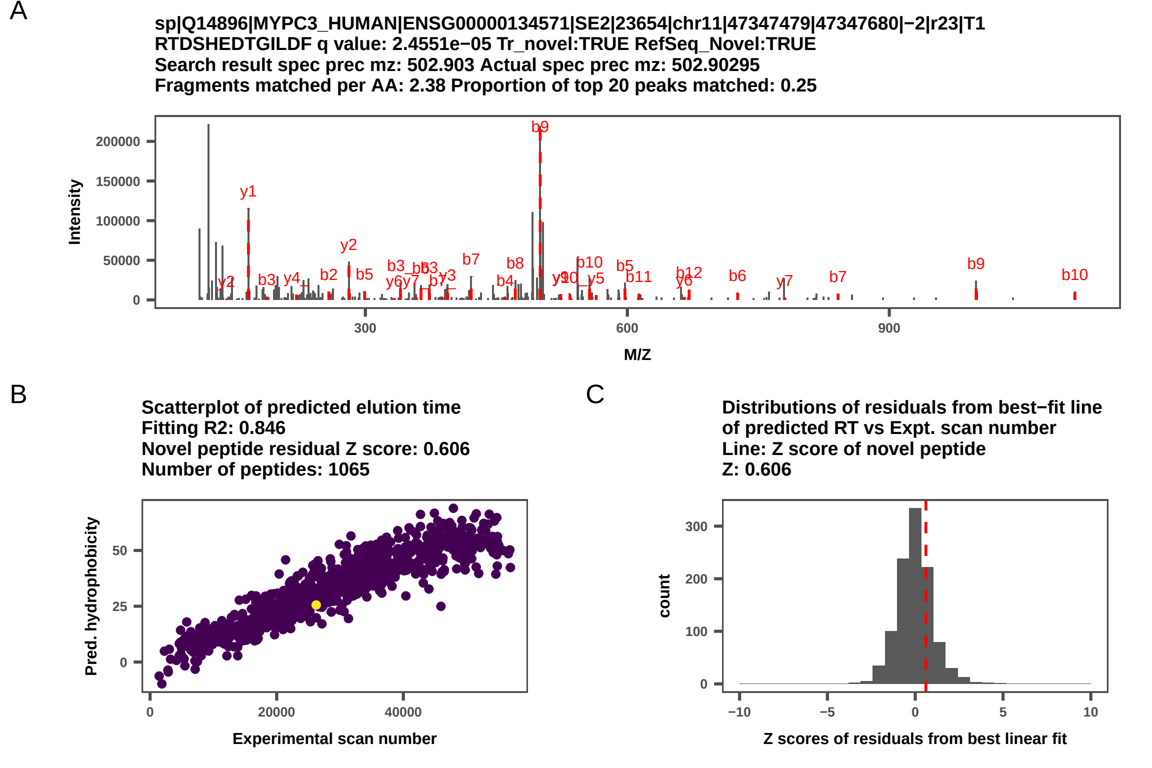
A
sp|Q14896|MYPC3_HUMAN|ENSG00000134571|SE2|23654|chr11|47347479|47347680|−2|r23|T1
RTDSHEDTGILDF q value: 2.4551e−05 Tr_novel:TRUE RefSeq_Novel:TRUE
Search result spec prec mz: 502.903 Actual spec prec mz: 502.90295
Fragments matched per AA: 2.38 Proportion of top 20 peaks matched: 0.25
Intensity
200000
150000
100000
50000
0
M/Z
300
600
900
y1
y2
b9
b2
b5
b7
b8
b10
b5
b11
b12
b6
b7
b9
b10
B
Scatterplot of predicted elution time
Fitting R2: 0.846
Novel peptide residual Z score: 0.606
Number of peptides: 1065
Pred. hydrophobicity
50
25
0
0
20000
40000
Experimental scan number
C
Distributions of residuals from best−fit line
of predicted RT vs Expt. scan number
Line: Z score of novel peptide
Z: 0.606
count
300
200
100
0
−10
−5
0
5
10
Z scores of residuals from best linear fit
